# Mediterranean and MIND Diets Containing Olive Biophenols Reduces the Prevalence of Alzheimer’s Disease

**DOI:** 10.3390/ijms20112797

**Published:** 2019-06-07

**Authors:** Syed Haris Omar

**Affiliations:** Rural Clinical School, Faculty of Medicine, University of New South Wales, Harvey House, Docker Street, Wagga Wagga, NSW 2650, Australia; haris.omar@unsw.edu.au or syedharisomar@gmail.com; Tel.: +61-2 6933-5140

**Keywords:** Alzheimer’s disease, cognitive decline, Mediterranean diet, olive biophenols

## Abstract

The risk of Alzheimer’s disease (AD) increases with nonmodifiable conditions including age and lack of effective efficacious pharmacotherapy. During the past decades, the non-pharmacotherapy mode of treatment of dietary modification received extensive attention in AD research. In order to reduce the AD pathology and cognitive decline, various dietary patterns have been attempted including caloric restriction (CR), dietary approaches to stop hypertension (DASH), ketogenic diets (KD), Mediterranean diet (MedDi) and Mediterranean-DASH diet Intervention for Neurological Delay (MIND) diet. Higher adherence to the MedDi diet was associated with decreases in cardiovascular and neurological disorders including AD and related cognitive decline. However, another emerging healthy dietary pattern MIND diet has also been associated with slower rates of cognitive decline and significant reduction of AD rate. Olive serves as one of the building block components of MedDi and MIND diets and the exerted potential health beneficial might be suggested due to the presence of its bioactive constituents such as oleic acids and phenolic compounds (biophenols). A few trials using medical food showed an optimal result in presymptomatic or early stages of AD. The review supports the notion that MedDi and MIND diets display potential for maintaining the cognitive function as nonpharmacological agents against AD pathology and proposed preventative mechanism through the presence of olive biophenols and presents the gaps along with the future directions.

## 1. Introduction

Brain ageing may be considered as a progressive and inevitable physiological process related to the dramatic increase in the oxidative stress condition and inflammatory markers that potentially cause damage to cellular structures of the brain [1]. The consequences of the long-term ageing of the brain results in speed and memory performance decline [1]. It has become evident that ageing is one of the major risk factors that strongly influence the instigation and progression of many neurodegenerative disorders including Alzheimer’s disease (AD), Multiple sclerosis, Parkinson’s, and Huntington’s diseases [1]. Neurodegenerative diseases affect people of all ages, however, AD is the most common form of dementias affecting people of usually over 65 years old and cause an increase in the global health challenge with 40–50 million people currently living with dementia [2]. AD is considered as a multifactorial and heterogeneous phenomenon involving aberrant protein processing called senile plaques and neurofibrillary tangles, composed of amyloid beta (Aβ) and paired helical filaments of hyperphosphorylated tau protein [3]. To date, there is no cure or treatment available to even alter the progressive course of AD. There are a few FDA approved drugs that regulate the activity of neurotransmitters (e.g., Memantine, an NMDA receptor antagonist; donepezil, a cholinesterase inhibitor) and showed some stabilization with the limited temporary and symptomatic support of cognitive functioning (mild to moderate stages) in these patients [4].

Following continuous attempts of using synthetic origin molecules against AD and their unfortunate failure in pharmacotherapy, the research trend has been changed towards using plants in diet or medication form that have positive effects on cognitive disorders, as well as strong acetylcholinesterase (AChE) inhibitory, anti-inflammatory, and antioxidant activities, and are of potential clinical interest for the treatment of AD [5]. Evidence amassed from numerous preclinical and clinical studies between 1990 and 2018, suggested a strong association link between a few dietary patterns and AD incidence [6], presenting diet as a modifiable risk factor that may play a central role in managing the illness [7]. Intervention in these areas may reduce the risk of developing AD or at least delay its clinical symptoms [8]. Dietary guidelines for the prevention of AD and healthy brain ageing recommend generous daily consumption of vegetables, whole grains, legumes, fruit, nuts and seeds [9].

Since 2000, it has become increasingly evident that biophenols, plant phenolic compounds containing foods received extreme attention due to their vast occurrence and versatile actions including several cellular functions modulation, processes that go well beyond their first-described natural antioxidant capacities and neuroprotection [10]. Vegetables, fruit, nuts, chocolate and other types of foods and beverages, such as wine, coffee and tea, are all rich sources of biophenols. 

The present review summarizes the existing evidences of adherence to diets (specially the Mediterranean diet (MedDi) and Mediterranean dietary approaches to stop hypertension (DASH) diet Intervention for Neurological Delay (MIND)) with the reduction in AD risk and cognitive function and exploring the involvement of olives as one of the backbone dietary components. In addition, the neurobiological mechanisms of the MedDi against AD or cognition decline are proposed.

## 2. Search Criteria and Data Collection

The published scientific literatures were searched for in vitro, in vivo, observational studies, prospective cohort studies and randomized controlled trials of adult human participants reporting diet/nutritional intake and AD biomarkers. The searches were conducted digitally by using the databases including MEDLINE, PubMed, ScienceDirect, Google Scholar, and SCOPUS to identify peer-reviewed articles in the last two decades (January 1, 2000–April 14, 2019). A Boolean search strategy was conducted with the following keywords and logic: (“diet” OR “dietary patterns” OR “Mediterranean diet” OR “MIND diet” OR “caloric restriction diet” OR “DASH diet” OR “ketogenic diet”) AND (“Alzheimer’s disease” OR “amyloid” OR “β amyloid” OR “β-amyloid” OR “Aβ42” OR “Aβ40”) AND (“olive biophenols” OR “olive polyphenols” OR “oleuropein” OR “hydroxytyrosol”). 

## 3. Dietary Pattern Attempted in Alzheimer’s Disease Prevention and Treatment 

Alzheimer’s disease can be influenced by several factors and the potential effect of diet has become a topic of increasing scientific and public interest. It was suggested that AD patients were associated with a high intake of meat, butter, high-fat dairy products, eggs, and refined sugar [11]. Animal studies showed that docosahexenoic acid (DHA) level altered cerebral haemodynamics (relative cerebral blood volume (rCBV)) in 8-month-old APP/PS1 and wild type mice, and cholesterol-containing typical Western diets (TWD) decreased rCBV in 15-month-old mice [12]. In addition, a short-term animal study showed that four weeks administration of Western diet was sufficient to selectively promote cerebral oxidative stress and metabolic disturbances in amyloid precursor protein and presenilin-1 (APP x PS1) knock-in mice, with increased oxidative stress preceding alterations in Aβ [13]. In Australian and Swedish studies [14,15], Western dietary pattern showed the consistent results of faster decline in cognitive abilities, however, the findings for prudent dietary patterns (mostly characterised by intake of fruit, vegetables, lean meat, fish and unprocessed grains) were inconsistent. It was observed that there is no associations of cognitive decline with higher scores on the Healthy Eating Index that is based on the U.S. Department of Agriculture (USDA) dietary guidelines [16], or for incident dementia with higher scores on the World Health Organization (WHO)-recommended diet or a low-carbohydrate diet pattern [17].

### 3.1. Calorie Restriction Diet

Caloric restriction (CR) dietary interventions influence the ageing and age-related changes in the brain and ultimately extend the lifespan and health span. In a transgenic mouse model of AD study [18], two different dietary energy restriction regimens including 40% CR and intermittent fasting (IF) protected against cognitive decline, however only the CR group have shown the lower levels of Aβ_40_, Aβ_42_ and phospho-tau in the hippocampus compared to the control diet group. Further, in a conditional double knockout of presenilin-1 and presenilin-2 (cDKO) mice study [19], administration of the CR diet for four months resulted in the improvement of cognitive impairments through the assessments of the novel object recognition test and contextual fear conditioning memory test as well as a reduction in the induction of tau hyperphosphorylation. In a recent animal study [20], the CR diet was associated with modest improvements in behavioural and cognitive outcomes, although the results were mainly limited to females and inconsistent.

A few earlier pieces of epidemiological evidence indicated that individuals who habitually consume fewer calories have a reduced incidence of AD [21,22]. In a randomized controlled trial [23], the effect of six months of CR (25% restriction) on overweight subjects did not show significant association with change in cognitive test performance (Table 1). In contrast, a trial on healthy elderly subjects [24], a CR (30% reduction) diet with the limitation of 1200 kcal/day for three months resulted in a significant increase in verbal memory scores (mean increase 20%; *p* < 0.001).

### 3.2. Assessment of Calorie Restriction Diet

Apart from the possible benefits of CR diets in the lowering of blood pressure and improved heart health to longevity, it was also suggested that a reduction up to 30 % of daily calorie intake can cause gaunt look or emaciated and experience dizziness, cognitive decline, loss of muscle mass, a faulty menstrual cycle and a lowered libido. Moreover, animal studies showed that eliminating more calories up to 60 % of daily intake resulted into signs of starvation in mice. 

The researchers asserted that long-term calorie restricted diet can increase stem cell functionality but may also reduce the performance of the immune system especially regarding bacterial infections. Although, there is experimental evidence for a calorie restricted diet as an ageing intervention, the potentially harmful effects and risks in elderly humans need to be studied further.

However, initiating a CR intervention prior to adulthood imposes many practical, physiological, and ethical challenges, including consideration of the impact on growth and development in humans. Therefore, introducing a CR intervention prior to adulthood is not an option in human studies.

### 3.3. Dietary Approaches to Stop Hypertension (DASH) Diet 

The dietary approaches to stop hypertension (DASH) diet mainly contains a high intake of plant foods, fruits, vegetables, fish, poultry, whole grains, low-fat dairy products, and nuts, alongside low intake of red meat, sodium, sweets, and sugar-sweetened beverages [30]. The DASH dietary pattern became popular in context to reduce cardiovascular health abnormalities, however there are only a few trials that have been conducted to show the beneficial health in cognition related AD. In a randomized trial, administration of the DASH diet showed the improvement in neurocognitive and psychomotor functions among older adults with high blood pressure (Table 1), at greater risk for cognitive decline and AD [25]. Moreover, a long-term study in older people showed slower rates of cognitive decline by 0.007 standardized units slower or equivalent to at least 4.4 years younger age after DASH diet administration, and suggested this was through an anti-inflammatory mechanism [26].

Coronary heart disease and hypertension are independent risk factors for AD [31], while few studies have reported an association between raised serum cholesterol levels and an increased risk of developing AD [32]. A randomized trial in patients randomly assigned to a DASH diet on corticosteroid therapy for 10 weeks showed significantly (*p* = 0.04) different systolic and diastolic blood pressures [33]. Moreover, diabetes has been recognized as a risk for development of AD [34], and the same randomized trial showed that serum total cholesterol and fasting blood glucose were significantly decreased in the corticosteroid medications patient following the DASH diet [33]. Studies have been suggested that high levels of low-density lipoprotein (LDL) cholesterol and total cholesterol (TC) concentrations were associated with an increased risk of AD [35], which may result in cognitive impairment. In a randomized crossover trial, DASH diet, but not the high fat-DASH diet, significantly reduced LDL cholesterol, HDL cholesterol, apolipoprotein A-I, intermediate-density lipoprotein and large LDL particles, and LDL peak diameter compared with the control diet [36]. 

### 3.4. Assessment of DASH Diet 

Despite the known benefits of the DASH diet, adherence has been limited to the general population. The approach taken for DASH diet constructing an adherence score is a major limitation to the study. Incomplete data on the DASH diet made it difficult to take into account specific intake levels for each food group according to energy requirements for individuals because these data were not available. Additional research is needed to determine whether this finding also holds true for men and other race/ethnicity, as well as in the educated, socioeconomic status subgroups.

### 3.5. Ketogenic Diet (KD)

A ketogenic diet primarily consists of high-fats (55%–60%), moderate-proteins (30%–35%), and very-low-carbohydrates (5%–10%) and provides 2000 kcal per day diet [37]. Administration of ketone bodies or high-fat, low-carbohydrate ketogenic diets (KD) may help in the augmentation and supply of brain fuel in later life [38], and the increasing number of animal and human studies data demonstrated the utility and usefulness of KD in AD. In a short-term animal study [39], a KD-fed group of transgenic mice exhibited low levels of both Aβ_40_ and Aβ_42_. In an animal study [40], dogs were administered (2 g/kg/day) medium chain triglycerides (MCT) which are rapidly converted to ketone bodies, for two months, showed dramatically improved mitochondrial function and decrease in total Aβ levels in the parietal lobe. Another animal study using transgenic mice [41], showed significant improvements in the learning and memory performance test after administration of a ketone ester-based diet (KET). In addition, KET-fed mice exhibited decreased Aβ deposition in the hippocampus and the amygdala along with reduction in hyperphosphorylated tau deposition in the hippocampus, amygdala, and cortex regions [41]. 

In a double-blind placebo-controlled study on AD patients [27], the oral dose of MCT facilitated performance on the Alzheimer’s Disease Assessment Scale-Cognitive Subscale (ADAS-cog) and was associated with an elevation in ketone body beta-hydroxybutyrate (β-OHB) levels (Table 1). In a randomized, double-blind, placebo-controlled, parallel-group study [28], administration of an oral ketogenic compound AC-1202 (10–20 g) resulted in significant improvement of the ADAS-cog test in AD patients compared to placebo. Short-term (six weeks) consumption of low carbohydrate (20 g/day) caused significant improvement of verbal memory performance in older adults with Mild Cognitive Impairment (MCI) [29].

### 3.6. Assessment of Ketogenic Diet

In summary, KD showed an improvement in cognitive outcomes, which was associated with the level and duration of ketosis [42]. In addition, improvement in the cognitive outcome was more pronounced in ApoE4-negative patients. It was also reported that the cognitive improvement was also found in elderly, non-demented people [43]. Currently, there is limited evidence of the efficacy of these diets in AD. In order to get a significant outcome, the ketogenic diets could be administered early in the presymptomatic stages of AD.

## 4. Promising Dietary Patterns Associated with the Prevention or Attenuation of AD

A number of studies have shown the slow rate of cognitive decline and reduced risk of cognitive impairment and suggested that the decreased rate of dementia have been associated with a higher consumption of single nutrients (e.g., vitamins B–E, tocopherol, polyunsaturated omega-3 fatty acids, biophenols) or of single foods (e.g., olive oil, vegetables, fruits, nuts, red wine, and fish [44,45,46,47,48]).

### 4.1. Mediterranean Diet

The theoretical concept of the Mediterranean diet was originally coined and documented by Ancel Keys [49], in the Seven Countries Study. However, the Mediterranean diet (MedDi) is characterized by a high intake of vegetables, legumes, fruits, and cereals; a high intake of extra virgin olive oil as unsaturated fatty acids, but low intake of saturated fatty acids; a moderately high intake of fish; a low-to-moderate intake of dairy products; a low intake of meat and poultry; and a regular but moderate amount of ethanol, primarily in the form of wine during meals [50]. Of the multiple components of the MedDi, fat reaches 40% of total calories, yet nearly all of this comes from olive oil. According to the USDA (United States Department of Agriculture), extra virgin olive oil contains 73.33 g of monounsaturated fatty acids, 13.33 g of saturated fatty acids, and 6.67 g of polyunsaturated fatty acids per 100 mL and considered as the best oil for consumption [51].

The MedDi, primarily known as a food model, enhances the quality and safety of foods and is accepted worldwide including Australia [52] due to multiple pharmacological effects and health benefits including cardiovascular disease prevention in populations inclusive of individuals with diabetes [53]. Research has suggested that the primary benefits of MedDi is due to richness in unsaturated fat and biophenols from the core component olive [54]. 

A systematic review showed higher adherence to MedDi was associated with better cognitive function, lower rates of cognitive decline, and reduced risk of AD in nine out of 12 studies [55]. Further meta-analysis of 22 studies showed the higher adherance to MedDi was consistently associated with reduced risk for cognitive impairment (RR = 0.60, 95% CI = 0.43–0.83) [56]. The data are consistent as well with large observational studies providing longitudinal evidence of a moderate protective effect of the MedDi against cognitive decline and AD. Since the last two decades, MedDi received higher attention and showed associated with a reduced risk of developing MCI and AD, and a reduced risk (33%) of progressing from MCI to AD [57]. In a Greek study [58], adherence to the MedDi was associated with better performance in memory, language, visuospatial perception and the composite cognitive score; the associations were strongest for memory in dementia and MCI patients. Meta-analysis of cohort studies revealed a significant association between MedDi and older adults’ episodic memory and global cognition, but not working memory or semantic memory [59]. Meta-analysis of RCTs revealed that compared with controls, MedDi improved delayed recall, working memory, and global cognition but not episodic memory, immediate recall, paired associates, attention, processing speed, or verbal fluency and strongly suggested the beneficial effect of the MedDi in healthy older adults’ global cognition [59]. Furthermore, a higher level of evidence from six RCTs and 31 cohorts studies, a recent systematic review [60] reported that the MedDi was the most investigated with evidence supporting protection against cognitive decline among older adults and suggested the mechanism through antioxidant activity from phenolic compounds and anti-inflammatory activity from omega-3 fatty acids containing MedDi. 

### 4.2. Assessment of Mediterranean Diet

Studies suggested the interactions and overlaps exist between diet and other lifestyle factors, such as physical exercise [61,62]. For example, a randomized controlled trial using a robust design conducted in highly active people in a Mediterranean culture demonstrated the cognitive benefits in participants that consumed MedDi [63]. Another major limitation and threat to external generalizability of studies on associations between cognitive functions and the MedDi is that there are no a priori determinations of cut-off points or recommendations regarding the exact composition of the MedDi.

### 4.3. MIND Diet

An observational study that combined two dietary plans including the MedDi and DASH diets called Mediterranean-DASH diet Intervention for Neurological Delay (MIND) diet (fifteen dietary components make up the MIND diet [64]), observed a 53% reduction in the rate of AD [65] and suggested that the MIND diet substantially slows cognitive decline with age [66,67]. Generally, the MIND diet differs from the MedDi by allocating separate categories for green leafy vegetables and berries, and a category for cakes and pastries. Unlike the MedDi, fruit was excluded, and fish was not prescribed daily because evidence suggested two to three times a week is adequate for neuroprotective effects [66]. In a recent study [68], MIND dietary pattern was associated with better cognition among the elderly subjects living in middle to low income countries.

### 4.4. Assessment of MIND Diet

Across four studies, the MIND diet has been associated with reduced AD risk [65] and better cognitive performance [69,70] but inconsistently with the cognitive decline [69]. To date, the cognitively protective effect of the MIND diet has not been evaluated or compared with the MedDi outside the United States; two of the four MIND diet studies were conducted in the same sample belonging to the Chicago-based Memory and Ageing Project (MAP). Clearly, trials of the MIND diet conducted in other populations and geographic locations are required to further evaluate its protective effects.

## 5. Proposed Mechanism of MedDi and MIND Diets Action against Alzheimer’s Disease

The exact pharmacological mechanism by virtue MedDi exerts a protective effect in brains are not fully understood. Despite the lack of studies investigating the underlying mechanisms, MedDi and MIND diets showed multiple pharmacological action including anti-oxidant, anti-inflammation, anti-atherogenic, and cognitive enhancement mediated through its food components. However, it may suggest that the health beneficial effects of the MedDi and MIND diet have been attributed to the use of olive as a major source of dietary fats and phenolic compounds [71].

### 5.1. Olive’s Major and Minor Components

**Figure 1:**
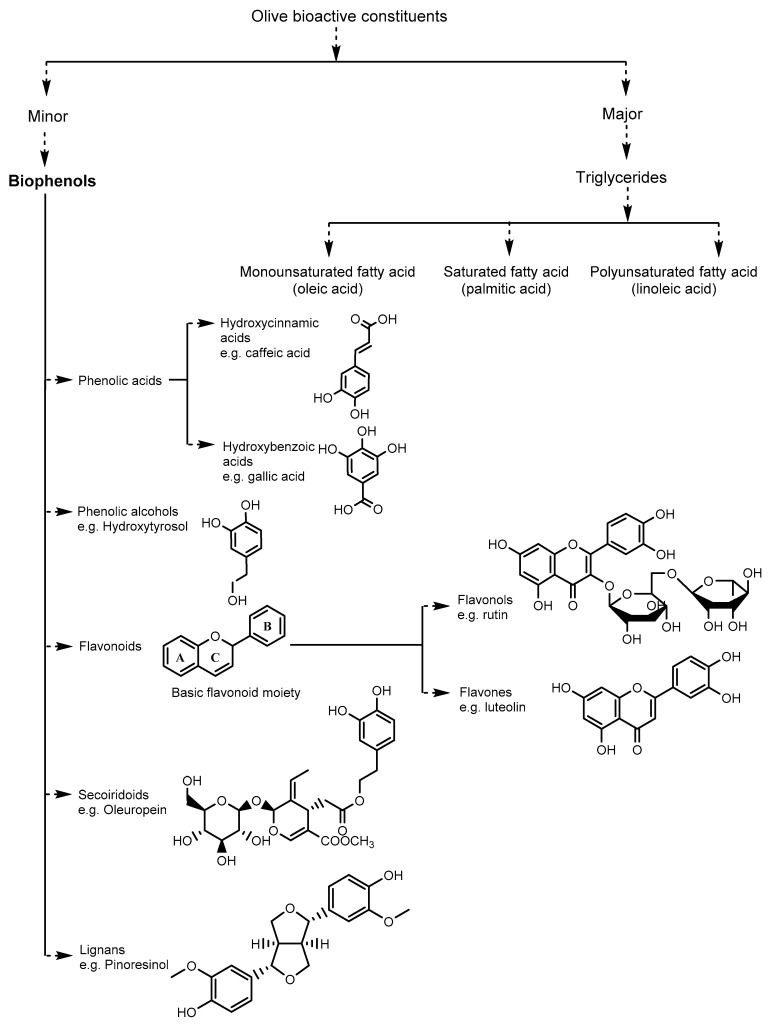
A few major and minor bioactive constituents in olive leaves, fruits, and oils.

Triglycerides are the major constituents of olive oil (98–99% by weight) categorized into three main fatty acids fraction including monounsaturated fatty acid (oleic acid), saturated fatty acid (palmitic acid) and polyunsaturated fatty acid (linoleic acid) respectively [72]. There is a plethora of non-polar unsaponifiable fraction present in olive oil as a minor fraction (approximately 2% of the weight), among them, phytosterol, squalene, tocopherols, sterols, and triterpenic compounds along with the polar phenolic compounds (Figure 1) [73]. 

Phenolic compounds called biophenols isolated from plant tissues or products are derived from shikimate-phenylpropanoid and/or polyketide pathway(s) including their derivatives, conjugates, degradation products and metabolites [74]. Olive contains a large variety of biophenols and their concentrations depend upon the types of oil as well as the source such as leaves > fruits > oils [75]. However, the occurrence of phenolic compounds in olives and olive oils are variable according to the olive variety, age of the tree, agricultural techniques used in cultivation, degree of ripeness, soil composition, climate, processing technique, and storage conditions [76]. 

In general, secoiridoids compounds, and tyrosol (*p*-HPEA) or its hydroxyl derivative hydroxytyrosol (3,4-DHPEA) are the most abundant class of phenolics found in all olive products. However, olive oil, specially the extra virgin olive oil (EVOO), is mostly represented by the presence of abundant secoiridoids as aldehydic forms of oleuropein and ligstroside aglucones (3,4-DHPEA-EA and *p*-HPEA-EA, respectively) [77]. In addition, lignins were concentrated as (+)-1-acetoxypinoresinol and (+)-1-pinoresinol in EVOO. The European Union regulation (EUn.432/2012) allows the acknowledgement for extra virgin olive oil containing more than 250 mg kg^−1^ of biophenols (hydroxytyrosol and its derivatives e.g., oleuropein complex and tyrosol) with the health claim “Olive oil polyphenols contribute to the protection of blood lipids from oxidative stress” [78]. Olive fruits mainly contain phenols as secoiridoids oleuropein and dimethyl oleuropein, and phenolic glycosides such as ligstroside, and hydroxycinnamic acid derivative verbascoside [79]. Olive leaves contain secoiridoids (oleuropein, ligstroside, dimethyloleuropein, and oleoside), flavonoids (apigenin, kaempferol, luteolin) and phenolic compounds (caffeic acid, tyrosol, hydroxytyrosol). 

**Figure 2:**
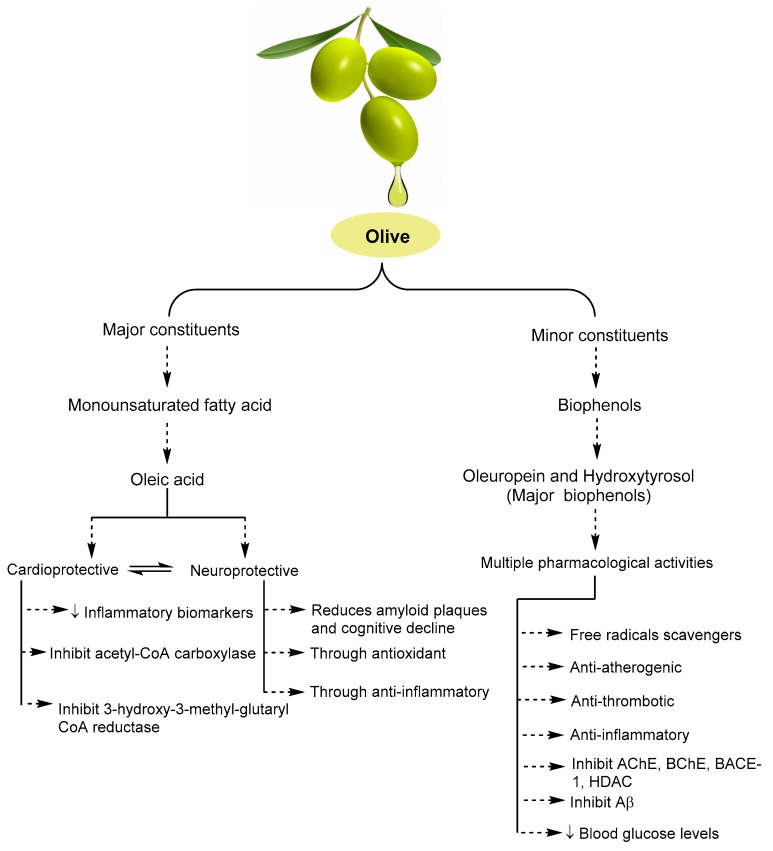
Some of the important pharmacological activities showed by olive’s major and minor constituents.

Biophenols serve as potent scavengers through capturing free radicals (Figure 2), combining with peroxyl and alkoxyl radicals, and chelate trace metals. Studies have suggested that the scavenging property seems to be more efficient in the decarboxymethyl and aldehydic forms of oleuropein aglycone compared with hydroxytyrosol [80]. It was suggested that oleuropein and its derivatives are better antioxidants than vitamin C, vitamin E, and the synthetic antioxidant butylated hydroxytoluene [81].

### 5.2. MedDi against AD Mediated through Olive’s Monounsaturated Fatty Acid

Dysregulation of unsaturated fatty acids including oleic acid metabolism plays a major role in driving AD pathology [82]. Oleic acid is the highest content of triglyceride as monounsaturated fatty acid (MUFA) in olive oil and are typically thought to be crucially responsible for the cardioprotective effects when compared with diets containing oils with a dominant content of saturated fatty acids [83,84]. A few studies have proposed the improvement in cognitive function mediated through the positive effects on cardiovascular risk factors [85,86]. In an elderly population of southern Italy study, administration of a typical MedDi was associated with a high level of protection against age-related cognitive decline (ARCD) suggested due the presence of MUFA [87]. A population-based prospective study in nondemented elderly (65–84 years) subjects consuming a typical MedDi containing high MUFA showed protective activity against ARCD [88]. In a recent study on two groups consuming MedDi and MedDi plus low dose (~26 g) extra virgin olive oil showed significant improvement in cognitive functions, suggested through its anti-oxidant and anti-inflammatory action in the brain [89]. 

Inflammation, a well-known causative factor of several diseases showed a significant association with compromised vascular health, as well as neuronal damage in the brain, through amyloid peptides accumulation and subsequent activation of microglia and reactive astrocytes [90]. Prevention of NLRP3 inflammasome activation [91], and cause downregulation of circulating inflammatory biomarkers [92], supported the beneficial effects through MUFAs in MedDi [93]. There is no direct role of high cholesterol in the pathogenesis of AD, however, a few studies have shown membrane cholesterol play a role in the formation and aggregation of Aβ [94], and concluded that high total cholesterol in midlife (>6.5 mmol/L) may increase the risk of AD but urge cohort studies to publish their data on this topic to increase the knowledge base [95]. Oleic acid from the olive in MedDi was found to be involved in the lowering of cardiovascular and neurological disorder through the mechanism of acetyl-CoA carboxylase (ACC) and 3-hydroxy-3-methyl-glutaryl CoA reductase (HMGCR) inhibition [96]. Oleic acid supplementation reduced secreted Aβ levels in amyloid precursor protein (APP) 695 transfected Cos-7 cells and reduced BACE-1 levels, presenilin levels, and reduced amyloid plaques in transgenic mice [97]. The in vitro and in vivo study reported anti-inflammatory and vasculo-protective activities of oleic acid and that it provided improvements in insulin resistance and type 2 diabetes mellitus [98]. There is a need of studies using oleic acid against various AD biomarkers and a description of the molecular mechanism of protection in humans.

### 5.3. MedDi Action against AD Mediated through Olive Biophenols 

As MedDi is a plant-based dietary pattern particularly rich in biophenols, usually olives and olive oil together provided approximately 11% of total biophenols intake, followed by red wine, which contributed approximately 6%. 

Multiple pharmacological activities (Table 2) have been associated with olive biophenols (oleuropein, hydroxytyrosol, verbascoside, and quercetin) as potent antioxidants [74], which can scavenge superoxide anion, hydrogen peroxide, hypochlorous acid [75], with anti-atherogenic, anti-thrombotic, anti-inflammatory properties [74], and with inhibition the enzymes involved in AD pathogenesis (acetylcholinesterases, butyrylcholinesterases, beta-secretases, histones deacetylases) [99], and reducing Aβ toxicity [100]. Similarly, MedDi biophenols have shown favourable effects in cognitive function through its anti-oxidant capacity [101] and anti-inflammatory properties [102]. MedDi containing biophenols, especially oleuropein and hydroxytyrosol, were significantly inhibited events connected with endothelial activation, including the expression of adhesion molecules such as VCAM-1, E-selectin, and to a lesser extent, ICAM-1 after stimulation with virtually any stimulus able to elicit the coordinated expression of such genes [103] through reduction of reactive oxygen species levels and nuclear factor (NF)-kB nuclear translocation and activation. 

Diabetes is an established link with cognitive decline, and olive biophenols showed antidiabetic effect through significantly improved insulin sensitivity and pancreatic β-cell secretory capacity [104]. However, there are still other possible mechanisms of brain protection by MedDi, such as an increase of neurotrophic factors related to neurotransmission, synaptic plasticity, and elimination of Aβ from the brain. A report from the three-year PREDIMED study showed the significant increase in plasma concentrations of brain-derived neurotrophic factor in the MedDi plus nuts group [105]. 

Overall, the findings provide insights into the proposed mechanisms of action of the MedDi containing olive biophenols in the context of prevention of neuroprotection and cognitive decline. Studies highlight significant cardioprotective and protection from age dependent cognitive decline which was suggested due the presence of oleic acid and biophenols in MedDi. Further experiments will involve the investigation into the effects of MedDi in aged and Alzheimer’s patients to provide insights into the potential anti-Alzheimer’s properties of the olive biophenols.

Growing evidence from the recent experimental and clinical data show the key role of gut dysbiosis and gut microbiota-host interactions in neurodegeneration, and may contribute in the pathogenesis of AD in elderly through the convergence of gut-derived inflammatory response together with ageing and poor diet [114]. 

Significant reduction of gut bacteria *Eubacterium rectale* and *Bacteroides* *fragilis* counts having anti-inflammatory action and rise of pro-inflammatory bacteria *Escherichia/Shigella* counts were observed in the cognitively impaired elderly patients with brain amyloidosis [115]. Studies have shown the positive correlation between MedDi dietary pattern and gut bacteria through the biophenol-induced increase in *Bifidobacteria* counts and anti-inflammatory activity with reduction in C-reactive protein and plasma cholesterol concentrations [116,117]. Further study supported the gut microbiota and dietary intervention approaches to modify the gut microbiota population specially through the consumption of biophenols which resulted in the increase of *Bifidobacteria* population [108].

There is a need of research regarding MedDi diet-microbiota and specific inflammatory biomarkers in AD, as there are not many studies that explore this connection. Moreover, probiotic therapy by using measured quantity of specific olive biophenols are also warranted which may offer an intriguing approach to promote host health via delivering anti-inflammatory mediators.

## 6. Trends of Medical Foods against Alzheimer’s Disease

Based on the recognized scientific principles and medical evaluation, a medical food is formulated to be consumed or administered under the direct supervision of physician and intended to meet specific dietary or nutritional requirement for management of a disease or condition. The products are considered medical foods in the European Union, however in the United States the products are regulated as dietary foods for special medical purposes. It was found that the symptomatic benefits in AD have been claimed for three medical foods (Table 3) including Axona, Souvenaid, and CerefolinNAC [118].

### 6.1. Axona

The dietary intake of coconut or palm kernel oils was suggested to be unable to provide caprylic triglyceride in sufficient quantities to meet the needs of people with AD [119]. Axona consists of medium-chain caprylic triglyceride, which was intended to be metabolized to ketones, and provided an alternative source of energy to the brain. The underlying rationale of Axona use was to diminish the glucose metabolism in the brain of AD patients and compensated by ketone bodies serving as an alternative source of energy. Axona was found to be helpful in the clinical dietary management of the metabolic processes related to mild-to-moderate AD [120]. However, due to presence of frequent adverse events, primarily transient, mild-to-moderate gastrointestinal effects, Axona was not approved by US Food and Drug Administration as a medical food for the AD therapy. Further, studies are warranted to determine the safety and clinical efficacy of Axona in AD patients.

### 6.2. Souvenaid

Souvenaid is a multinutrient beverage enriched with 11 vitamins and supplements, approved for early AD in some European countries and Australia. Souvenaid formulation consists of phosphatide precursors along with the supporting nutrients, which may intend to act synergistically to enhance the membrane formation and synaptic function improvement in individuals with AD [121]. In addition, the beverage mixture contains omega-3 fatty acids eicosapentaenoic acid and docosahexaenoic acid, vitamins B6, B12, C and E, choline, folic acid, selenium, uridine monophosphate, and phospholipids at levels above to those contained in the normal diet. To date, no further studies have been investigated to verify the 12-week effects of Souvenaid in patients with mild AD. Indeed, long-term study using Souvenaid in phospholipid formation, synaptic function, and cognitive abilities in people with and at risk of AD need to be examined in randomized controlled trials with a large sample size and over substantially longer periods of time.

### 6.3. CerefolinNAC

CerefolinNAC is a mixture of L-methylfolate, methylcobalamin, and N-acetyl-cysteine, mainly to address the metabolic imbalances of hyperhomocysteinemia and neurovascular oxidative stress involved in the cognitive impairment. Administration of CerefolinNAC in AD or cognitive impaired patients having hyperhomocysteinemia due to cerebrovascular disease resulted in an adjusted hippocampal atrophy rate 4.25 times slower than those with AD and related disorders but not those with hyperhomocysteinemia who did not receive CerefolinNAC [122].

However, the study has not been further verified and needs to be investigated in the future in a large sample size with a longer follow-up period and a randomized assignment to the CerefolinNAC treatment and placebo. Moreover, there is a need to emphasize that the findings in individuals with hyperhomocysteinemia cannot be extrapolated to those with normal homocysteine levels.

## 7. Conclusions

**Figure 3:**
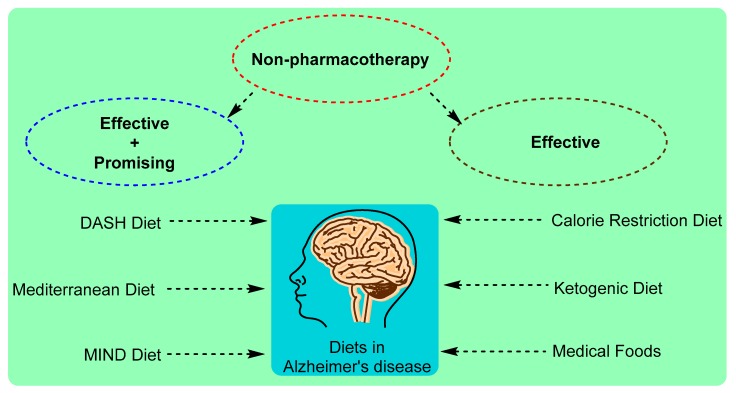
The non-pharmacotherapy of AD through dietary modification.

The neuroprotective activity of olive phenolic constituents including oleuropein, hydroxytyrosol, verbascosides, and oleocanthal are well documented [100,124,125,126,127]. According to the European Food Safety Authority (EFSA), hydroxytyrosol is the only phenolic compound that has received a health claim approval [128], and suggested that 5 mg of HT and its derivatives (e.g., oleuropein and tyrosol), provided by moderate amounts of olive oil, can be easily consumed in the context of a balanced diet, although the concentration of some olive oils may be too low to achieve this intake. However, the specific activity in AD with cognitive decline still need to be investigated from each of the olive biophenols. As olive biophenols represented one of the key ingredients in the MedDi and MIND diet, the health beneficial effects due to their presence may be speculated too (Figure 3). Studies have shown the adherence to the MedDi associated with a reduced risk for coronary heart diseases and metabolic syndrome including hypertension and dyslipidemia, which have been associated with the development of cognitive impairments [129,130]. 

In summary, accumulated evidence from animal studies, epidemiological studies, and clinical trials linked higher adherence to a MedDi to a reduced risk of cognitive decline and dementia. However, the prescription of the MedDi as a preventive or therapeutic measure in AD is hampered by the lack of established levels of individual dietary components, especially of bioactive phytochemicals whose identification, content, and efficacy are well characterized.

## 8. Future Direction

The health benefits of olive’s major and minor constituents i.e., oleic acid and biophenols are well established in context to cardioprotective and neuroprotective activity. Furthermore, it is also well documented that the most important health-promoting mechanisms induced by a MedDi are: a lipid-lowering effect, protection against oxidative stress, inflammation and platelet aggregation, modification of hormones and growth factors involved in cancer, the inhibition of nutrient sensing pathways by restricting specific amino acids, and gut microbiota-mediated production of metabolites [131].

However, the cognitive benefits of MedDi and MIND diet are not very well investigated in context to the use of olive or olive biophenols. Following considerations are warranted:

- The need to stick with a uniform source of olive intake (virgin or extra virgin olive oil or fruit) in the MedDi and MIND diet.
- The measured portion or percentage of olive or olive biophenols are missing in the MedDi and MIND diet. There is a need for a specified and uniform amount of olive inclusion in both diets.

Indeed, there is a need to investigate the randomized trial in placebo versus MedDi or MIND diet group which may include or exclude olive in cognitive decline patients.

## Figures and Tables

**Table 1:** Clinical studies on dietary pattern associated with the risk of Alzheimer’s disease.

Dietary Model	Study Type	Outcomes	References
Calorie restricted	Caloric restriction (25%) alone or with physical exercise (6 months)	Daily energy deficit was not significantly associated with change in cognitive test performance	[23]
Calorie restricted	Caloric restriction (30% reduction) diet with limitation of 1200 kcal/day for 3 months	Significant increase in verbal memory scores (mean increase 20%; *p* < 0.001)	[24]
Calorie restricted (DASH Diet)	Caloric restriction with physical exercise (4 months)	Participants on the DASH diet combined with a behavioural weight management programme exhibited greater improvements in executive function memory-learning (*p =* 0.008) and psychomotor speed (*p* = 0.023) compared with the usual diet control	[25]
DASH Diet	DASH diet was administered in older people	DASH score was associated with a slower rate of global cognitive decline by 0.007 standardized units (standard error of estimate = 0.003, *p* = 0.03)	[26]
Ketogenic Diet	Medium chain triglycerides (MCT) in AD patients	Facilitated performance on the (ADAS-cog) Subscale and associated with rise in ketone bodies	[27]
Ketogenic Diet	Oral administration of ketogenic compound AC-1202 (10–20 g) in AD patients	Significant improvement in the ADAS-cog	[28]
Ketogenic Diet	Administration of carbohydrate (5–10%) per day in older adults with MCI	Significant improvement in verbal memory performance	[29]

**Table 2:** Major pharmacological activities of olive biophenols.

Activity	Study	Action	References
Antioxidant	In vitro	Verbascoside, oleuropein, and caffeic acid scavenges superoxide radical and protected H₂O₂-induced SH-SY5Y cells	[5,75]
Cardioprotective	Randomised controlled trial	OLE reduces blood pressure and plasma TC, LDL-C and TAG	[106]
	Randomised controlled trial	OLE improve vascular function and reduce inflammatory cytokine (IL-8)	[107]
	Randomised controlled trial	Virgin olive oil phenolic compounds cause increase in *bifidobacteria* population together with increase in biophenols microbial metabolites	[108]
Neuroprotective	In vivo	Oleuropein aglycone reduces Aβ_42_ deposition, plaque deposit, and improves the cognitive performance	[109]
	In vitro	Oleocanthal inhibited aggregation of tau protein	[110]
	In vitro and in vivo	Oleuropein and hydroxytyrosol reduces Aβ_42_ aggregation in SH-SY5Y cells and plaques formation in mice	[100]
Metabolic syndrome	In vivo	Hydroxytyrosol decrease fasting glucose level in obese mice	[111]
	Randomised controlled trial	Olive oil phenolic compounds improve endothelial function in hypertensive patients	[112]
	Randomised controlled trial	OLE improve insulin sensitivity and pancreatic β-cell secretory capacity	[104]
Enzyme modification	In vitro	Olive mill waste phenolic compounds increase GSH levels in erythrocytes	[113]
	In vitro	Olive biophenols inhibited AChE, BChE, BACE-1 and HDAC	[99]

Aβ: amyloid beta; AChE: acetyl cholinesterase; BACE-1: β-secretase; BChE: butyryl cholinesterase; HDAC: histone de-acetylase; LDL-C: low density lipid cholesterol; OLE: olive leaf extract; TAG: triacylglycerol; TC: total cholesterol.

**Table 3:** Significance of medical foods in Alzheimer’s disease (AD).

Brand Name	Ingredients	Mechanism of Action	Significance	References
Axona	Caprylic triglyceride	Caprylic acid to ketone bodies metabolism provided an alternative energy source for neurons with compromised glucose utilization	Cause significant increase in serum ketone bodies 2 h after administration. A significant difference was found between Axona and placebo groups in mean change from baseline in ADAS-cog score on days 45 and 90 of administration (Axona group improving and controls worsening)	[28]
Souvenaid	Omega-3 fatty acids (eicosapentaenoic acid, Docosahexaenoic acid), vitamins B6, B12, C, and E, choline, folic acid, selenium, uridine monophosphate, phospholipids	Effects on deficits in neuronal membrane composition and function as well as improvement of synapse formation	A significant improvement was found in delayed verbal recall and unchanged clinical outcomes after daily administration for 12 weeks.	[121]
			A significant improvement in memory domain function was found and assessed with a neuropsychological test battery	[123]
CrerefolinNAC	L-methylfolate, methylcobalamin, N-acetyl-cysteine	Effects on metabolic imbalances and neurovascular oxidative stress in hyper-homocysteinemia	A significant decrease in hippocampal and cortical atrophy rates were found in participants with both AD and hyperhomocysteinemia	[122]
